# Cardiac injury caused by iron overload in thalassemia

**DOI:** 10.3389/fped.2025.1514722

**Published:** 2025-01-27

**Authors:** Chunxi Fu, Xue Yang

**Affiliations:** ^1^ Department of Pediatrics, West China Second University Hospital, Sichuan University, Chengdu, China; ^2^ Key Laboratory of Birth Defects and Related Diseases of Women and Children, Ministry of Education, Sichuan University, Chengdu, China

**Keywords:** β-thalassemia, iron overload, myocardial injury, treatment, early identification

## Abstract

Cardiac iron overload affects approximately 25% of patients with β-thalassemia major, which is associated with increased morbidity and mortality. Two mechanisms are responsible for iron overload in β-thalassemia: increased iron absorption due to ineffective erythropoiesis and blood transfusions. This review examines the mechanisms of myocardial injury caused by cardiac iron overload and role of various clinical examination techniques in assessing cardiac iron burden and functional impairment. Early identification and intervention for cardiac injury and iron overload in β-thalassemia have the potential to prevent and reverse or delay its progression in the early stages, playing a crucial role in its prognosis.

## Introduction

β-thalassemia is the most common inherited disease, characterized by decreased or absent β-globin chain synthesis and hemoglobin A production (1–3). An estimated 1.5% of the global population is reported to be β-thalassemia carriers (4). It is most common in individuals from or ancestry from African countries, the Indian subcontinent, the Mediterranean, the Middle East, and Southeast Asia (1–6). In recent years, the prevalence of β-thalassemia in Europe and the North America has been on the rise, largely attributed to immigration patterns (7). β-thalassemia can be categorized into non-transfusion-dependent thalassemia (NTDT) and transfusion-dependent thalassemia (TDT) based on the level of reliance on blood transfusions (8). According to a 10-year retrospective cohort study, mortality rates for TDT in England were 6.2%, significantly higher than the age/sex-adjusted mortality rate of 1.2% for the general population (9).

The incidence of myocardial iron overload in transfusion-dependent β-thalassemia patients has increased from 11.4%–15.1% in early studies to 26.1%–36.7% in recent studies (10, 11). This may be due to increased survival leading to a higher rate of comorbidities (12, 13). Cardiovascular disease remains the primary cause of death among patients with β-thalassemia, while iron overload persists as a significant challenge (14). Two mechanisms are responsible for iron overload in β-thalassemia: increased iron absorption due to ineffective erythropoiesis and blood transfusions (15). Due to ineffective red blood cell production, NTDT patients experience anemia and hypoxia, which suppresses hepcidin expression, thereby promoting the absorption of iron in the intestine (16, 17). Furthermore, low levels of hepcidin will cause an upregulation of transferrin, further promoting the excessive iron release by macrophages (18). TDT patients receive blood transfusions, which equates to an average daily intake of approximately 0.40 mg/kg iron. For patients who receive 25–30 U red blood cells per year, the cumulative iron in the patient will exceed 70 g by the 30th year (8, 19).

As iron loading progresses, the capacity of transferrin to bind and detoxify iron is eventually exceeded, leading to non-transferrin-bound iron in plasma, which promotes oxidative stress, mitochondrial dysfunction, and ferroptosis (20). Myocardial iron overload can cause iron overload cardiomyopathy (IOC), which presents as restrictive or dilated cardiomyopathy, heart failure (HF) (21–23), and atrial fibrillation (AF) (24, 25). Because the outcomes of transfusion-dependent β-thalassemia are often driven by cardiac involvement, early diagnosis and monitoring are crucial for patient management.

### Mechanisms of myocardial injury caused by iron overload

Studies have shown that iron myocardial overload causes myocardial dysfunction. Unbound iron enters the cardiomyocytes through the L-type calcium channels (26). Endosome-mediated uptake might also be involved (27). Once in the cardiomyocytes, iron binds to ferritin and is transported to lysosomes for degradation and long-term storage (27). When oxidative stress overcomes the defenses, the rapid Fenton reaction leads to an overproduction of hydroxyl ions, an extremely reactive ion that will participate in lipid peroxidation and change membrane permeability. Such permeability can cause a leak of hydrolytic enzymes that initiate cell damage and culminate in cardiomyocyte death (28). In the presence of iron overload with concomitant ischemia, iron overload will exacerbate ischemia-reperfusion injury (29, 30). Unbound iron continuously entering the heart and vasculature aggravates the damage and pathological processes caused by iron overload and other heart pathologies (e.g., previous myocardial ischemia) (31, 32). Iron overload in the context of ischemia/reperfusion injury will also aggravate injury through ferroptosis (33–36). Disruptions in calcium ion metabolism by iron overload in cardiomyocytes are thought to contribute to cardiac dysfunction in such patients (37), while heart fibrosis does not appear to be involved (38). These may also be mechanisms by which cardiac iron overload causes cardiac dysfunction in thalassemia patients.

The fact that cardiomyocyte iron overload is a storage problem and not an infiltrative process indicates that iron can be removed to reverse cardiac iron overload and associated damage (39). Combination iron chelation therapy for severe transfusional myocardial iron overload reduced liver iron overload but without changes in left ventricular ejection fraction (40). On the other hand, Khamseekaew et al. (41) showed that iron chelation therapy led to decreased cardiac oxidative stress, improved cardiac mitochondrial function, and improved cardiac function. The increased cardiac risk could also be due to other metabolic abnormalities induced by iron overload, including insulin resistance (32, 42).

### Molecular mechanism of myocardial injury caused by cardiac iron overload in thalassemia

**Figure 1:**
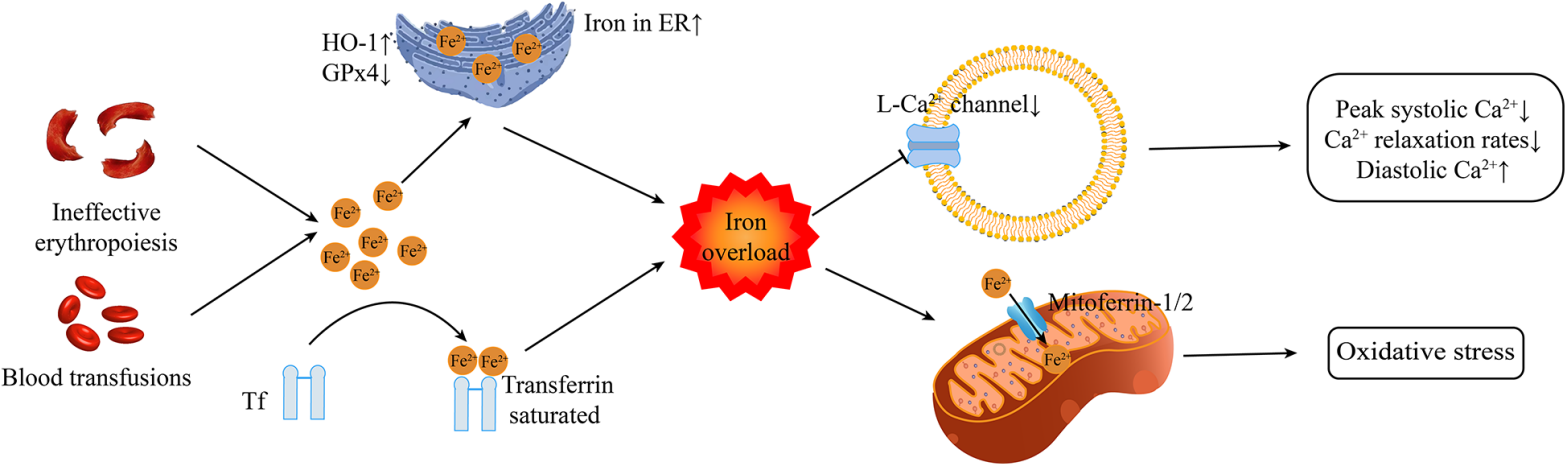
The primary mechanisms of cardiac injury associated with iron overload Tf transferrin. ER, endoplasmic reticulum; HO-1, heme oxygenase-1; GPx4, glutathione peroxidase 4.

Iron overload is a common feature in patients with β-thalassemia, but the molecular mechanism of cardiac damage caused by iron overload in thalassemia is poorly understood. The following has been reported so far. Figure 1 outlines the main cardiac injury mechanisms associated with iron overload.

#### Iron metabolism

At present, the exact molecular mechanism of iron overload leading to heart damage is mainly considered to be ferroptosis (33–36). Ferroptosis is a form of cell death dependent upon intracellular iron and is distinct from apoptosis, necrosis, and autophagy (43). Heme oxygenase 1 up-regulation in response to hypoxia and hypoxia/reoxygenation degrades heme and induces or exacerbates iron overload and ferroptosis in the endoplasmic reticulum of cardiomyocytes. Ferroptosis triggered by GPx4 reduction and iron overload in the endoplasmic reticulum completely differs from mitochondria-driven necrosis (33) and will participate in cardiomyocyte death (44). Mitochondrial iron uptake depends on the activity of the mitochondrial Ca^2+^ uniporter, which is essential in causing mitochondrial dysfunction and ferroptosis in cardiac iron overload (34).

Hepcidin, a peptide hormone originated from the liver, regulates iron absorption and distribution by suppressing the activity of transferrin (45). The transferrin is accountable for modulating iron absorption from the gastrointestinal tract and its release in macrophages and hepatocytes. Additionally, iron ions binding to transferrin significantly reduce its potential toxicity, while unbound free iron ions are the main factor causing tissue cell damage (46, 47). Transferrin is saturated by iron overload in β-thalassemia (48). Under the stimulation of iron overload and inflammation, the expression of Hepcidin is upregulated; whereas under the stimulating conditions of anemia, hypoxia, or the synthesis/injection of erythropoietin, its expression is downregulated (49–51). Nevertheless, in patients with β-thalassemia, the generation of hepcidin is decreased due to ineffective erythropoiesis (52–54). In patients with thalassemia, the high levels of growth differentiation factor 15 (GDF15) and erythroferrin can also inhibit the production of hepcidin (55, 56). This gives rise to a vicious cycle: hepcidin levels in patients with β-thalassemia are frequently too low, thereby triggering excessive iron absorption in the intestines and the mobilization of iron in macrophages, ultimately leading to iron overload in various organs, including the heart (52–54). Regular blood transfusions are likely to inhibit the expression of hepcidin driven by erythropoiesis in patients with TDT (57). Consequently, the hepcidin level in TDT patients is typically higher than that in NTDT patients (57). In addition to the regulatory role of hepcidin, the expression of iron transporters such as transferrin receptor 1 (Tfr1) and transferrin is crucial for understanding the dynamics of cardiac iron overload (48). Tfr1 mediates the uptake of iron bound to transferrin, while transferrin facilitates the excretion of iron from cells (58, 59). In β-thalassemia, disruption of these transporters, coupled with low hepcidin levels, leads to an imbalance in iron metabolism, further exacerbating iron accumulation in cardiac tissue (60).

The therapy targeting the hepcidin-transferrin axis has demonstrated initial potential, capable of effectively alleviating iron overload and ineffective erythropoiesis. The specific mechanisms and therapeutic effects will be elaborated on in detail in the treatment section that follows.

#### Calcium channel

Calcium is a critical regulator of cardiac function by maintaining cardiac excitation-contraction coupling, and disturbances in cardiac calcium regulation are a major contributor to left ventricular dysfunction in iron overload cardiomyopathy (37). L-type Ca^2+^ channels participate in iron uptake into cardiomyocytes (61). Iron overload reduces CaV1.3-dependent L-type Ca^2+^ currents (24). In the presence of elevated oxidative stress, such as in iron overload, sarcoplasmic reticulum Ca^2+^ leaks through the Ca^2+^ release channels (62), sarcoendoplasmic reticulum Ca^2+^-ATPase activity is inhibited, the sodium-calcium exchanger currents are elevated (63), and L-type calcium channels are reduced (64, 65). Reduced peak systolic Ca^2+^ levels, slow Ca^2+^ relaxation rates, and elevated diastolic Ca^2+^ levels contribute to impaired cardiomyocyte functions (66). L-type Ca^2+^ channels are also found in pancreatic beta cells (67, 68) and the parathyroid gland cells (69). Therefore, iron overload will also induce endocrine dysfunction that can indirectly influence the heart.

T-type calcium channels also play a significant role in the pathophysiology of myocardial iron overload, particularly in patients with β-thalassemia. In conditions of iron overload, such as those seen in thalassemia, T-type calcium channels may serve as critical pathways for iron entry, exacerbating cardiac iron accumulation and contributing to cardiomyopathy. Research has demonstrated that T-type calcium channels are involved in the mechanisms of iron uptake in cardiomyocytes. Specifically, iron overload conditions have been shown to increase the expression and activity of T-type calcium channels, leading to enhanced iron influx into the cardiomyocytes. This process is particularly concerning because excessive iron deposition can lead to oxidative stress, mitochondrial dysfunction, and ultimately, heart failure. The interplay between calcium and iron metabolism is complex, as elevated intracellular calcium levels can further promote iron-induced cellular damage, creating a vicious cycle of injury in the myocardium (70, 71).

Atrial and ventricular tachyarrhythmias can develop due to heterogeneous electrical conduction and repolarization (72). Iron overload reduces CaV1.3-dependent L-type Ca^2+^ currents, resulting in atrial fibrillation (24). Sudden cardiac death may occur without involvement of ischemia or infarction (73–75). These are enough to show that the risk of cardiac iron overload can affect the life safety of patients with thalassemia.

#### Oxidative stress

In β-thalassemia, ineffective erythropoiesis results in increased hemolysis and the release of free hemoglobin, which further exacerbates oxidative stress through the Fenton reaction, where iron catalyzes the conversion of hydrogen peroxide into highly reactive hydroxyl radicals (76). Furthermore, the accumulation of malondialdehyde, a marker of oxidative stress, has been observed in patients with β-thalassemia, indicating heightened oxidative damage (77). The role of hypoxia-inducible factor 1-alpha (HIF-1a) in this context is particularly noteworthy. HIF-1a is known to be involved in cellular responses to hypoxia and oxidative stress. In cases of iron overload, HIF-1a levels may be altered, influencing the expression of genes involved in iron metabolism and oxidative stress response (78). This dysregulation can lead to increased erythroid apoptosis and further exacerbate the iron overload condition, creating a vicious cycle of damage (78). The accumulation of ROS is particularly detrimental in cardiac tissues, where it can disrupt calcium homeostasis, contributing to cardiac dysfunction and arrhythmias (79).

Glutathione (GSH), a key antioxidant in the body, plays a vital role in mitigating oxidative stress. In thalassemia patients, the dysregulation of the glutathione antioxidant system can exacerbate the effects of iron overload. Studies have shown that patients with thalassemia exhibit reduced levels of GSH, which compromises their ability to neutralize ROS effectively (80, 81). This reduction in GSH levels is often correlated with increased markers of oxidative damage, such as malondialdehyde and total carbonyls, indicating a higher degree of lipid peroxidation and protein oxidation (82).

The upregulation of glutamate-cysteine ligase, an enzyme involved in GSH synthesis, has been observed in some thalassemia patients as a compensatory response to oxidative stress. However, this adaptive mechanism may not be sufficient to counteract the overwhelming oxidative burden caused by iron overload (83). The interplay between iron accumulation and the glutathione antioxidant system is crucial, as the depletion of GSH can lead to further cellular damage and contribute to the progression of cardiac complications in thalassemia patients.

Moreover, the use of antioxidants has been explored as a potential therapeutic strategy to mitigate oxidative stress in thalassemia patients. For example, alpha lipoic acid has shown promise in reducing oxidative stress markers and improving iron levels in β-thalassemia major patients (84). Similarly, vitamin E supplementation has been associated with decreased oxidative stress, suggesting that antioxidant therapy could play a role in managing myocardial iron overload and its associated complications (85). The combination of deferiprone (an iron chelator) and N-acetylcysteine (an antioxidant) has been reported to enhance cardiac calcium homeostasis and reduce oxidative stress in iron-overloaded thalassemic mice (86).

Other pathways could also be involved in cardiac injury due to iron overload. The nuclear erythroid factor-2 (Nrf2) is a transcription factor involved in redox responses and modulates anti-inflammatory and cytoprotective systems to ensure cell survival against oxidation in different tissues (87). Still, Nrf2-deficient mice display age-dependent cardiomyopathy (87).

In summary, the mechanism of oxidative stress related to cardiac iron overload in β-thalassemia patients involves the interplay of excess iron, increased ROS production, and the resultant cellular damage. Addressing this oxidative stress through targeted therapies may improve clinical outcomes and reduce morbidity associated with cardiac complications in these patients.

#### Mitochondria

The pathophysiology of IOC in β-thalassemia patients is closely related to mitochondrial dysfunction, which can be further exacerbated by iron overload in cardiac tissue. The mitochondria are central to energy production in all cells and are affected by iron overload, leading to mitochondrial oxidative stress and participation in ferroptosis (34). The excess cytoplasmic iron leads to excess oxidative stress (88). Iron can enter the mitochondria through the mitoferrin −1 and −2 proteins, damaging the mitochondria through oxidative damage and decreasing energy output (41, 88).

Recent studies have highlighted the role of mitochondrial calcium uniporters (MCU) in mediating iron-induced mitochondrial dysfunction. Research indicates that iron overload can lead to increased ROS production, which in turn disrupts mitochondrial membrane potential and promotes cell death pathways such as ferroptosis. For instance, a study demonstrated that blocking MCU significantly reduced ROS production and mitochondrial swelling in iron-overloaded thalassemic mice, suggesting that MCU could be a therapeutic target for preventing mitochondrial dysfunction in IOC (41, 89). Moreover, the interplay between iron metabolism and mitochondrial dynamics is critical. Iron overload has been shown to impair mitochondrial biogenesis and dynamics, leading to altered mitochondrial morphology and function. In a murine model of iron overload, the expression of mitochondrial fusion and fission proteins was significantly disrupted, correlating with increased oxidative stress and cardiac dysfunction (41, 89). This disruption in mitochondrial dynamics is further compounded by the activation of apoptotic signaling pathways, which are triggered by elevated iron levels and ROS (90). The role of signaling pathways such as the PI3K/AKT/mTOR and MAPK pathways in mediating the effects of iron overload on mitochondrial function has also been explored. For example, icariin, a natural compound, has been shown to protect bone marrow mesenchymal stem cells from iron overload-induced dysfunction by modulating these pathways, thereby enhancing mitochondrial function and reducing apoptosis (91). This suggests that targeting these signaling pathways may provide a novel approach to mitigate the effects of iron overload on cardiac mitochondria.

#### Genetics factors

Polymorphisms and mutations in specific genes can participate in cardiac iron overload. Mokhtar et al. (80) reported that glutathione S-transferase gene polymorphism (GSTM1) null genotype was associated with cardiac iron overload independent of serum ferritin in Egyptian patients with β-thalassemia. On the other hand, Abo-Shanab et al. (81) stated that the GSTM1 null genotype is not involved in β-thalassemia or cardiac complications. Hepcidin and hemochromatosis protein polymorphisms appear to be involved in iron homeostasis in patients with β-thalassemia (92, 93). Polymorphisms in the OPG/RANK/RANKL axis might also be involved in cardiac iron overload (94).

## Identify patients at risk for cardiac iron overload

### Magnetic resonance imaging

Cardiac magnetic resonance imaging (CMRI), especially T2*CMRI, is an excellent non-invasive detection method for identifying iron deposits within the heart (95, 96). Iron disrupts magnetic inhomogeneities and accelerates signal decay, thereby reducing T2* relaxation (97). T2* CMRI is a crucial marker for diagnosing and monitoring iron chelation therapy in IOC patients, significantly improving the survival of β-thalassemia major (β-TM) (98–100). T2* < 20 ms is considered to indicate iron overload in the myocardium, while T2* < 10 ms is an important predictor of IOC heart failure progression (101). The first CMRI should be performed as soon as possible after starting iron chelation therapy (102), as CMRI can guide iron chelation therapy (103, 104).

Due to T2* measurements being taken from the left ventriclar (LV) mid-ventricular septum, attention has increasingly shifted towards assessing iron loading and functionality in other cardiac chambers, such as the left atrium (LA) and right ventricle (105). LA strain may serve as an indicator of left ventricular diastolic function in patients with thalassemia, given that diastolic dysfunction often precedes contractility dysfunction (106–108). The study showed that LA strain parameters (including LA reservoir strain, LA conduit strain, and LA booster strain) were independently associated with cardiac complications in the β-TM cohort, stronger than cardiac iron levels (108). Iron overload can also directly damage the LA wall, promoting atrial cardiomyopathy and atrial fibrillation (109, 110).

### Echocardiographic

Cardiac iron overload does not have characteristic findings on echocardiography, and studies have shown that the left ventricular ejection fraction (LVEF) measured by echocardiography is not significantly correlated with the T2* measured by CMRI (111). Echocardiography has significant advantages in non-invasiveness, convenience, and wide application, and is suitable for follow-up monitoring of disease progression and therapeutic efficacy assessment in patients at high risk of iron overload or with confirmed iron overload. In addition, echocardiography can identify β-thalassemia patients with normal T2* MRI results but already showing impairment of cardiac diastolic function (111). A recent study suggested the potential value of echocardiography radiomics in predicting cardiac problems due to iron overload (112), which could be of value in settings where MRI is less available.

In β-TM patients, echocardiography showed that individuals with inter-atrial electromechanical delay >44.8 ms had 81.2% sensitivity and 98.7% specificity in identifying occult AF (113). Therefore, it is recommended to conduct long-term electrocardiographic monitoring to more effectively identify AF and prevent stroke (113). Moreover, the Left Atrioventricular Coupling Index (LACI) and Right Atrioventricular Coupling Index (RACI) are not related to myocardial iron load, but they are significantly increased in patients with cardiac complications (114).

### Electrocardiographic

As a basic diagnostic tool in clinical practice, the electrocardiogram (ECG) may also provide important clues as to whether iron overload is present (101). The prolongation of QRS duration, QT interval, and QTc interval is associated with cardiac iron overload in patients with transfusion-dependent thalassemia (115). Repolarization abnormalities and bradycardia serve as specific biomarkers in β-TM patients, aiding in the stratification of cardiac risk. Moreover, the electrocardiograms of β-TM patients without heart failure reveal inverted T waves and bundle branch blocks in about 46% of cases. AF occurs in 14% to 20% of cases (113, 116). The maximum *P*-wave duration and *P*-wave dispersion serve as effective ECG markers for the identification of AF occurring during a five-year follow-up in patients with β-TM (113, 116).

### Biomarkers

A study demonstrated that levels of growth differentiation factor-15, galectin-3, and N-terminal pro-B-type natriuretic peptide were significantly elevated in patients with myocardial iron overload compared to the healthy control group (117). However, these biomarkers did not exhibit a correlation with T2* CMRI and failed to predict myocardial iron overload in asymptomatic children with β-thalassemia major (117). Therefore, there are currently no biomarkers for diagnosis or guidance of treatment.

## Treatment

### Iron chelation treatment

Despite the novel comprehension of the pathophysiology of β-thalassemia, iron chelation therapy still remains the most efficacious approach for reducing the morbidity and mortality associated with iron overload in patients with NTDT and TDT (118). Currently, there are three commonly utilized iron chelators: deferoxamine (DFO), deferiprone (DFP), and deferasirox (DFX) (3). DFO is administered via subcutaneous or intravenous injection and can effectively lower hepatic iron concentration and patient mortality (3). Regarding cardiac iron overload, studies have demonstrated that a high-dose regime of continuous infusion for 24 h at 60 mg/kg/day can effectively reduce the cardiac iron burden and reverse cardiac complications (119, 120). Nevertheless, due to the requirement of prolonged parenteral administration, patients' compliance with DFO is limited (121). DFP is the first oral iron chelator and has been approved for use in TDT patients with contraindications to or insufficient response from DFO treatment. Owing to its lipophilic nature, DFP is more prone to enter cardiomyocytes, thereby reducing the iron load within cardiomyocytes (122). In comparison with DFO, patients treated with DFP exhibit more pronounced improvement in cardiac T2* values (123), higher left ventricular ejection fraction (123, 124), stronger cardiac protective effects (125), and a higher 5-year survival rate without heart disease (122). It is necessary to be cautious of the most severe adverse reactions, namely neutropenia and agranulocytosis, when using DFP for a long term (126). DFO, DFP, and DFX have all been shown to be effective for TDT patients (118). Based on the results of the THALASSA trial (127), DFX has become the only iron chelator approved for use in NTDT. For patients with myocardial T2* ≥ 20 ms, DFX treatment can prevent myocardial iron accumulation and be accompanied by an increase in LVEF (128). After 3 years of continuous DFX treatment, 68.1% of patients with a baseline T2* ranging from 10 to <20 ms had their T2* normalized, and 50.0% of patients with a baseline T2* ranging from >5 to <10 ms had their T2* elevated to 10 to <20 ms (129). Nevertheless, in the majority of studies, no alteration of LVEF was witnessed in patients with T2* < 20 ms who underwent DXP treatment (128–130). DFP is typically well tolerated, with adverse reactions such as elevated creatinine, abdominal pain, and nausea usually being transient and fleeting (131). The new film-coated tablet of DFX can conspicuously enhance patients' satisfaction and compliance with treatment (132).

Due to the differences in the iron chelation mechanisms of different iron chelators, some scholars believe that the combination of iron chelators may enhance the iron chelation effect through synergistic action (133, 134). Clinical studies have shown that compared with the use of DFO or DFP alone, the combination of DFO and DFP can more effectively reduce cardiac iron burden, improve cardiac function, and further increase the survival rate of patients (135–137). The combination therapy of DFP and DFX as well as that of DFO and DFX have both demonstrated superior efficacy compared to monotherapy, and no increase in drug toxicity has been observed (138–140). Currently, the combined therapy of DFP and DFO chelation is the most commonly used combined treatment for the treatment of severe cardiac iron overload (101). Nevertheless, at present, the selection of different combination treatment regimens still requires further exploration through head-to-head studies (141).

### Target hepcidin-ferroportin axis therapy

The therapeutic regimens targeting hepcidin can lower the risk of iron overload by suppressing iron absorption and potentiate the efficacy of iron chelation therapy. Minihepcidins are synthetic peptides encompassing 7–9 amino acids at the N-terminal of hepcidin, being capable of binding to iron transporter proteins and inducing their degradation (142). In the mice model, minihepcidins can effectively reduce iron load and improve ineffective red blood cell generation (43, 143, 144). However, the study evaluating the efficacy and safety of LJPC-401 (NCT03381833), a minihepcidin, for treating myocardial iron overload in TDT patients was prematurely terminated due to the lack of efficacy demonstrated in the interim analysis.

The inhibitor of transmembrane protease serine 6 (TMPRSS6) is capable of effectively reducing the degradation effect of TMPRSS6 on hepcidin (145). The anemia condition can be significantly ameliorated and the generation of ineffective red blood cells as well as the iron load can be reduced by knocking out the TMPRSS6 gene (146). It has been demonstrated that the RNA interference therapeutic agent targeting TMPRSS6 can effectively ameliorate anemia and iron overload in mouse models (147). IONIS TMPRSS6-LRx, functioning as an inducer of hepcidin, is capable of effectively alleviating the issue of iron overload (148). Currently, its therapeutic efficacy and safety in the human body are being assessed in Phase 2 clinical trials (NCT04059406).

Another approach is to employ iron transporter protein inhibitors (VIT-2763) to restriction the availability of iron, thereby mitigating ineffective erythropoiesis and iron overload (149). In the mouse model of β-thalassemia, oral administration of VIT-2763 can significantly enhance erythropoiesis and rectify the dysregulation of iron homeostasis. VIT-2763 in combination with DFX is also feasible, which provides a new opportunity to improve the ineffective red blood cell generation and iron overload in patients with β-thalassemia (150). The Phase 1 clinical study of VIT-2763 demonstrated that serum iron levels and transferrin saturation declined transiently, and no obvious safety concerns were identified (149). Currently, the Phase 2 clinical study of VIT-2763 (NCT04364269) is in progress.

Targeting the hepcidin-ferroportin axis offers a novel therapeutic direction for patients with β-thalassemia, particularly for those with NTDT, as it not only reduces iron overload but also improves anemia, decreases ineffective erythropoiesis, and alleviates splenomegaly. However, the impact of this therapeutic approach on cardiac iron overload requires further investigation.

#### Calcium channel blockers

Despite the role of calcium dysregulation in cardiac dysfunction induced by iron overload (37), Sadaf et al. (151) showed that the use of calcium channel blockers was not associated with improvements in cardiac iron overload. However, a preliminary study involving 26 patients with thalassemia who had received blood transfusions demonstrated that the combination of amlodipine and iron chelators could enhance cardiac T2* values, whereas no significant improvement was observed in the group receiving iron chelators alone (152). Subsequent randomized controlled trials further validated this finding; however, the combination of amlodipine and iron chelators did not lead to an improvement in LVEF (153–156). The clinical application of calcium channel blockers for the prevention and treatment of iron overload still requires more clinical randomized controlled trials to support it.

#### Decreases transfusion burden

Drug therapy has shown rapid progress in recent years. Drug therapy can promote red blood cell maturation, improve anemia, thereby reducing the need for blood transfusions and iron load. Thalidomide has been reported to increase hemoglobin levels or decrease blood transfusion volume by ≥50% in 78% of children with β-thalassemia and partial or declining response to hydroxyurea (157–159). Luspatercept is a recombinant fusion protein that binds to select transforming growth factor-beta (TGF-beta) superfamily of ligands (blocks SMAD2/3 signaling) to promote erythroid maturation and decreases transfusion burden and iron intake, lowering the requirement for iron-chelation therapy (160–162). Mitapivat is an allosteric activator of pyruvate kinase that reduces the markers of ineffective erythropoiesis and improves anemia (163). However, more clinical data is needed to evaluate the efficacy and safety of drug therapy. Luspatercept is a recombinant fusion protein that binds to select transforming growth factor-beta (TGF-beta) superfamily of ligands (blocks SMAD2/3 signaling) to promote erythroid maturation and decreases transfusion burden and iron intake, lowering the requirement for iron-chelation therapy (160–162).

### Anti-oxidants treatment

Recent research indicates that antioxidant treatment might exert a key role in controlling this situation by alleviating the oxidative stress related to iron overload. For example, DFP and NAC monotherapy showed similar cardioprotective effects, however, the combination of DFP with NAC showed more significant effects in reducing cardiac iron deposition and cell apoptosis compared to monotherapy (59). Some studies have explored the efficacy of different antioxidant therapies compared to traditional iron chelation methods. Research has shown that antioxidant compounds such as vitamin E (85, 164), vitamin C (165), silymarin (166), and NAC (167, 168) have a certain effect in reducing oxidative stress in cardiac tissue, and no serious side effects have been observed. Moreover, ebselen is a novel glutathione peroxidase mimetic, exerting anti-inflammatory and antioxidant effects (169). Ebselen combined with DFX treatment can significantly reduce the iron burden-induced cardiac hemosiderosis, cardiac malondialdehyde and improve cardiac function in mice with Mediterranean anemia (170). In the Phase II clinical trials, ebselen has demonstrated excellent tolerance and no severe adverse reactions (171). In conclusion, although iron chelation therapy remains the cornerstone of controlling cardiac iron overload in patients with β-thalassemia, the combination of antioxidant therapy as an adjunctive method shows great potential. Future studies should further explore the optimal combination of these treatments and their long-term impact on the cardiovascular health of this vulnerable population.

#### Gene therapy

Gene therapy is a potentially curative treatment for transfusion-dependent thalassemia (4). It included allogeneic hematopoietic stem cell transplantation (HSCT) and xenogenic stem cell suppression.

HSCT is the only definitive possible cure for patients with β-TM (2–4, 172), while the utility of HSCT in β-thalassemia intermedia is unclear (172). HSCT aims to provide stem cells with normal globin genes that would restore effective hematopoiesis. A successful HSCT can allow the management of iron overload by periodic phlebotomy rather than iron chelation therapy (1). Santarone et al. (173) reported an overall survival of >80% and disease-free survival of 74.5% in patients with β-TM 39 years after undergoing allogeneic HSCT. Di Bartolomeo et al. (174) showed that the 20-year overall survival was 89% and 20-year disease-free survival was 86% after HLA-matched HSCT in patients with β-TM, most of whom were considered high risk for transplantation-related complications. Still, despite its promises, HSCT has limitations related to age and issues with HLA matching (175–178). No randomized trials evaluated allogeneic HSCT in patients with β-TM found in a Cochrane review (179). HLA-matched unrelated donor and HLA-matched related donor HSCT are associated with similar event-free survival but HLA-matched unrelated donor HSCT may increase risk of acute and chronic GVHD in patients with β-TM (180). HLA-matched sibling donor HSCT may increase 2-year overall and event-free survival and reduce risk of extended chronic GVHD compared to other types of donor HSCT in patients with β-TM (181). Haploidentical HSCT reported to achieve 96% 3-year overall survival and 96% event-free survival in patients with severe β-thalassemia (182).

Xenogenic stem cell suppression is based on the use of autologous hematopoietic stem cells to overcome complications that can be a limiting factor in allogeneic HSCT, such as donor availability, risk of infections, graft rejection, and graft-vs.-host disease (4). The gene therapy products include betibeglogene autotemcel, exagamglogene autotemcel, and autologous CD34 + cells encoding the beta-A-T87Q-globin gene (but withdrawn from the European market in 2021) (183–193). Betibeglogene autotemcel is an autologous hematopoietic stem cell-based gene therapy that may increase functional adult HbA and total Hb to normal levels and eliminate dependence on regular packed red blood cell transfusions (185). Gene therapies, though promising, are still under development. Autologous transplantation of CD34 + hematopoietic stem and progenitor cells transduced with BB305 lentiviral vector encoding beta^A−T87Q^-globin gene was reported to eliminate the need for blood transfusion in 91% of children and adults ≤50 years old with TDT and nonbeta^0^/beta^0^ genotype (185). LentiGlobin BB305 vector gene therapy has been reported to reduce or eliminate the need for blood transfusions in patients with TDT (184). Exagamglogene autotemcel (exa-cel, formerly CTX001) CRISPR-Cas9 gene therapy was reported to increase fetal hemoglobin production through suppression of BCL11A expression and transfusion-independence in a 19-year-old female adult with TDT (beta^0^/beta^+^ genotype) in a case report (183).

### Anti-heart failure treatment

At the moment, there are a number of randomized controlled trials aimed at assessing the efficacy of pharmacological interventions, device-based therapies, or cardiac transplantation in β-thalassemia patients with HF. The treatment of β-thalassemia patients suffering from HF should be guided by guideline-directed anti-heart failure therapies. In an earlier cohort study involving 52 β-thalassemia patients with HF, who received chelation therapy in combination with standard anti-heart failure treatment had a survival rate of approximately 50% over a 5-year period, comparable to other HF patients (194). A recent study evaluated the mortality rates of patients with β-thalassemia and HF during two periods, 1992–2004 and 2004–2016 (195). The findings indicated a significant reduction in the proportion of deaths attributable to cardiac causes (195). Additionally, patients who achieved effective cardiac iron removal demonstrated sustained normal cardiac function over an average follow-up duration of 10 years (195). For patients with end-stage HF, heart transplantation is a worthwhile treatment option. Prior studies encompassed 16 recipients of IOC heart transplants from 1967 to 2003, reporting a 5-year survival rate of 81% (196). In cases where patients present with both end-stage heart failure and end-stage liver failure, it is advisable to consider combined heart-liver transplantation, as isolated organ transplantation may not yield improved prognostic outcomes (196).

### Management of atrial fibrillation

Currently, there is a lack of clinical studies investigating anti-coagulation or anti-platelet therapy in β-thalassemia patients with AF. Patients with β-thalassemia have a higher risk of stroke than the general population, which may be due to multiple factors such as iron overload, splenectomy, and hemolysis (101, 197). However, there is currently no stroke risk scoring system for β-thalassemia patients, and the CHA_2_DS_2_-VASc scoring system developed for the general population may not be applicable to these patients (13). Direct oral anticoagulants (DOACs) have the advantage of being easier to manage and safer than warfarin, but so far only a small-scale study has found that patients taking either DOACs or warfarin did not experience any ischemic or bleeding events during follow-up (198, 199). Furthermore, the risks of anemia and bleeding associated with β-thalassemia continue to pose significant challenges; thus, further clinical research is essential to inform anticoagulation decisions for these patients and to evaluate the safety profiles of DOACs in comparison to warfarin.

For the control of AF rate and rhythm, iron chelation therapy can effectively alleviate arrhythmia (40, 119, 200). When antiarrhythmic drugs must be used, there are no clinical studies specifically targeting the particularities of β-thalassemia patients. It is currently believed that drugs that may cause arrhythmias (such as flecainide and sotalol) should be used with greater caution, and non-dihydropyridine calcium channel blockers should be avoided in patients with HF (13). Amiodarone, a drug that effectively controls arrhythmias, may be relatively safe for short-term use. However, it is important to note that long-term use of amiodarone can cause damage to the thyroid, liver, and lungs, which are also the main organs affected by iron overload (201).

## Conclusion

Despite significant progress in the management of cardiac iron overload in β-thalassemia patients, cardiovascular disease remains the leading cause of death in this population. By gaining a deeper understanding of iron overload and its complex mechanisms in the heart, accurately assessing new therapeutic approaches based on chelators will help further improve patient outcomes. The roles and intervention timing of calcium ion channel antagonists, hepcidin activators, antioxidants, and gene therapy are currently under further investigation. If myocardial iron overload can be diagnosed and treated in the early stages of the disease, it will effectively prevent progression of the disease and improve survival rates. As previously mentioned, ECG, echocardiography, and CMRI all have their own advantages and limitations, and they complement each other. Utilizing current diagnostic methods together can help us detect and monitor iron deposition in the hearts of β-thalassemia patients in an early stage. However, observers may exhibit inconsistencies in the interpretation of results from CMRI, echocardiography, or ECG evaluations due to variations in their experience and expertise during the assessment (113, 202). In the contemporary landscape, advancements in artificial intelligence and machine learning have reached unprecedented levels, facilitating the effective integration of multidimensional data and the detection of subtle changes that may elude visual observation (202). Additionally, there is a relative lack of large-sample, high-quality studies in β-thalassemia patients to identify definitive treatment decisions. Federated learning can train artificial intelligence models using multi-center data while ensuring data privacy.
